# Implementation of Nanopore sequencing as a pragmatic workflow for copy number variant confirmation in the clinic

**DOI:** 10.1186/s12967-023-04243-y

**Published:** 2023-06-10

**Authors:** Stephanie U. Greer, Jacquelin Botello, Donna Hongo, Brynn Levy, Premal Shah, Matthew Rabinowitz, Danny E. Miller, Kate Im, Akash Kumar

**Affiliations:** 1 MyOme Inc., 535 Middlefield Rd Suite 170, Menlo Park, CA USA; 2 Department of Pathology and Cell Biology, Columbia University Irving Medical Center, New York, NY USA; 3 Natera Inc., San Carlos, CA USA; 4 Department of Pediatrics, Department of Laboratory Medicine and Pathology, University of Washington, WA Seattle, USA

**Keywords:** Long-read sequencing, Oxford Nanopore Technologies, Adaptive sampling, Copy number variants, Neurodevelopmental disorders, Clinical testing, Genome analysis, Targeted sequencing

## Abstract

**Background:**

Diagnosis of rare genetic diseases can be a long, expensive and complex process, involving an array of tests in the hope of obtaining an actionable result. Long-read sequencing platforms offer the opportunity to make definitive molecular diagnoses using a single assay capable of detecting variants, characterizing methylation patterns, resolving complex rearrangements, and assigning findings to long-range haplotypes. Here, we demonstrate the clinical utility of Nanopore long-read sequencing by validating a confirmatory test for copy number variants (CNVs) in neurodevelopmental disorders and illustrate the broader applications of this platform to assess genomic features with significant clinical implications.

**Methods:**

We used adaptive sampling on the Oxford Nanopore platform to sequence 25 genomic DNA samples and 5 blood samples collected from patients with known or false-positive copy number changes originally detected using short-read sequencing. Across the 30 samples (a total of 50 with replicates), we assayed 35 known unique CNVs (a total of 55 with replicates) and one false-positive CNV, ranging in size from 40 kb to 155 Mb, and assessed the presence or absence of suspected CNVs using normalized read depth.

**Results:**

Across 50 samples (including replicates) sequenced on individual MinION flow cells, we achieved an average on-target mean depth of 9.5X and an average on-target read length of 4805 bp. Using a custom read depth-based analysis, we successfully confirmed the presence of all 55 known CNVs (including replicates) and the absence of one false-positive CNV. Using the same CNV-targeted data, we compared genotypes of single nucleotide variant loci to verify that no sample mix-ups occurred between assays. For one case, we also used methylation detection and phasing to investigate the parental origin of a 15q11.2-q13 duplication with implications for clinical prognosis.

**Conclusions:**

We present an assay that efficiently targets genomic regions to confirm clinically relevant CNVs with a concordance rate of 100%. Furthermore, we demonstrate how integration of genotype, methylation, and phasing data from the Nanopore sequencing platform can potentially simplify and shorten the diagnostic odyssey.

**Supplementary Information:**

The online version contains supplementary material available at 10.1186/s12967-023-04243-y.

## Background

Copy number variants (CNVs)—losses (deletions) or gains (duplications) of segments of DNA—are a highly prevalent form of genomic variation, comprising between 4.8 and 9.5% of the genome [1]. CNVs have gained attention for their potential contribution to unexplained neurodevelopmental disorders (NDDs), such as developmental delay, intellectual disability, and autism spectrum disorder [2–7]. Given the variety and high prevalence of NDDs, the large number and frequent identification of genes involved, and the diversity in the type and size of pathogenic CNVs, improved methods for the molecular characterization of CNVs are needed to fill the gaps in our understanding of their impact on neurodevelopment.

In clinical testing, chromosomal microarray (CMA) is generally used to confirm results from short-read WGS CNV detection [8]. However, long-read sequencing can be used to accurately identify genomic aberrations and has the potential to improve diagnostic rates and turnaround times [9–11]. This technology improves mapping certainty and de novo assembly, while enabling detection and phasing of rare and clinically relevant variants, including CNVs, single nucleotide variants (SNVs), short tandem repeat (STR) expansions, and methylation differences, as well as refinement of both isolated and complex structural variant (SV) breakpoints [9, 12, 13]. Nanopore technology is increasingly flexible and capable of rapidly detecting variants invisible to short-read sequencing; however, it currently suffers from lower overall base-pair accuracy and increased cost compared to traditional short reads [12]. Adaptive sampling, a computational method for selecting and sequencing DNA molecules in real time, can be used to achieve adequate coverage of genomic regions of interest while reducing per-sample costs [10].

**Fig. 1:**
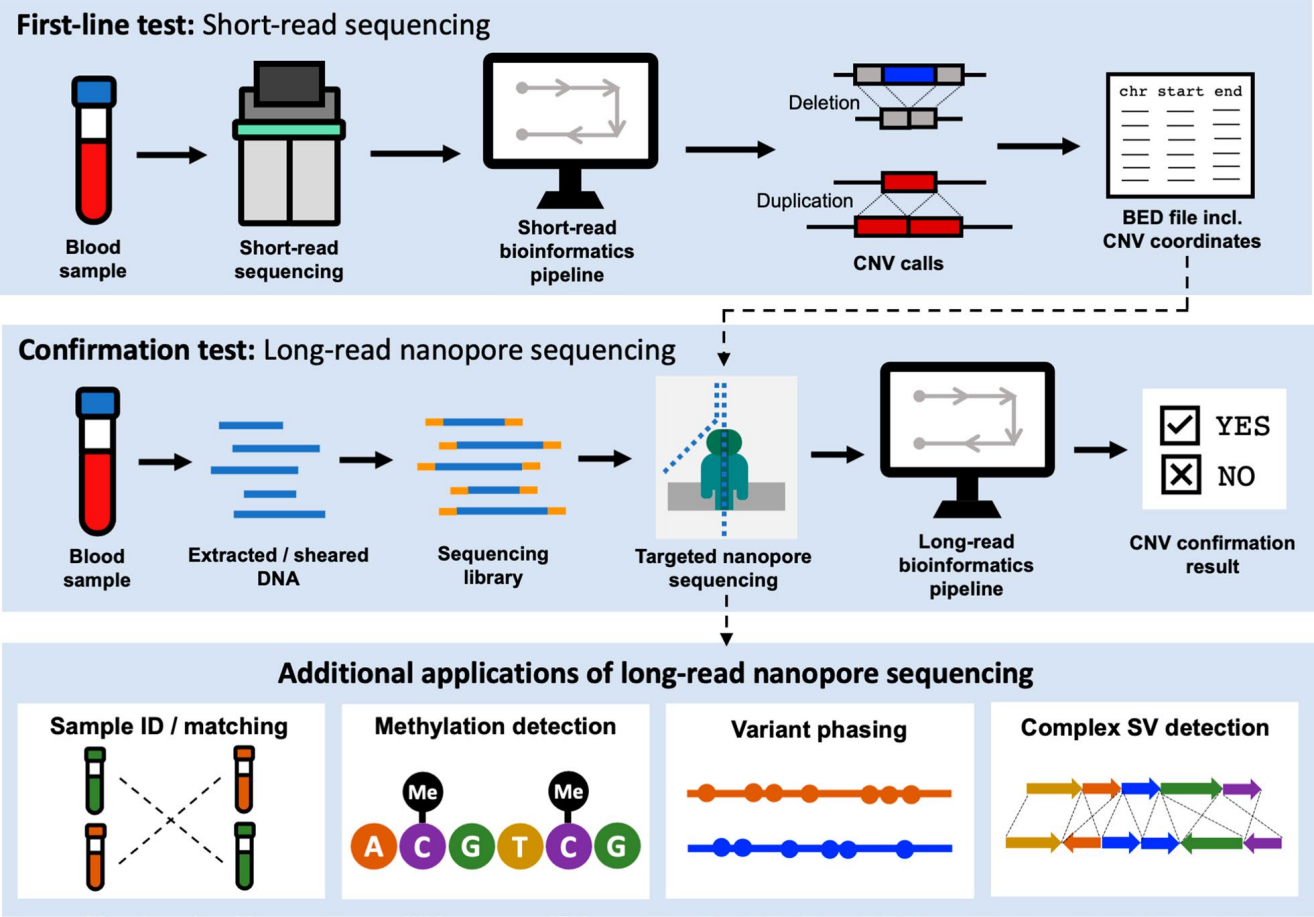
Overview of the Oxford Nanopore Technologies (ONT) copy number variant (CNV) confirmation assay. CNV coordinates are initially detected with short-read sequencing and then targeted and confirmed using ONT adaptive sampling along with a custom bioinformatics pipeline

We developed and validated a clinical test that uses Oxford Nanopore Technologies (ONT) long reads to confirm CNVs greater than 50 kb in size (Fig. 1). The performance of our test was validated using samples with known CNVs. For each sample, we used ONT adaptive sampling to target the suspected CNV region and then applied a custom bioinformatics pipeline to perform a read-depth analysis. Adaptive sampling enables real-time selection of DNA molecules in user-specified genomic target regions, which generates adequate on-target depth using a single flow cell for each sample. This test can be used as a clinical assay to confirm CNVs in samples from patients with NDDs.

## Methods

### Sample selection

We obtained 24 samples from individuals with known CNVs and emulated a false positive CNV by using one “normal” sample as a control (NA24385) from the Coriell Institute for Medical Research (Camden, New Jersey, United States) (Table 1 and Additional file 1: Tables S1 and S2). To test the ability of our assay to confirm a wide spectrum of CNVs, we selected samples with deletions and duplications of varying sizes, ranging from 40 kb to 155 Mb (Additional file 1: Table S3). We evaluated at least one known CNV in each Coriell sample and used a normal Coriell sample as a “false-positive” sample (Additional file 1: Table S2). We also collected samples from five de-identified healthy individuals known to carry a single CNV (Table 1 and Additional file 1: Table S2). All samples previously underwent whole genome sequencing on the Illumina platform, and CNVs were identified from the short-read data (internal results). The location and type of CNV identified from the short-read data served as controls in the present study. We tested 35 unique CNVs (55 with replicates) and emulated a false-positive case by looking for a CNV in a region known to be present in two copies in a normal Coriell sample (Table 1 and Additional file 1: Table S3). Personnel who performed the sample preparation and analysis were blinded to sample identity.

**Table 1:** Overview of samples and copy number variant (CNV) counts included in study

Sample num.	Sample ID	Sample source	Replicate sample count	False-positive CNV count	Known CNV count	Known CNVs	Known CNV count (incl. replicates)
1	NA24385	Coriell gDNA	1	1			
2	NA02894	Coriell gDNA	1		1	57-kb del	1
3	NA04520	Coriell gDNA	1		1	88-kb del	1
4	NA05090	Coriell gDNA	1		1	63-kb del	1
5	NA06936	Coriell gDNA	1		1	13-Mb del	1
6	NA10989	Coriell gDNA	1		1	12-Mb del	1
7	NA13554	Coriell gDNA	1		1	42-kb del	1
8	NA02422	Coriell gDNA	1		1	Trisomy 18	1
9	NA02948	Coriell gDNA	1		1	Trisomy 13	1
10	NA05124	Coriell gDNA	1		1	756-kb dup	1
11	NA05966	Coriell gDNA	1		1	21-Mb dup	1
12	NA09326	Coriell gDNA	1		1	XYY	1
13	NA09367	Coriell gDNA	1		1	35-Mb dup	1
14	NA17867	Coriell gDNA	1		1	XXY	1
15	NA22397	Coriell gDNA	1		1	5-Mb dup	1
16	NA06918	Coriell gDNA	1		2	212-kb del 17-Mb del	2
17	NA07945	Coriell gDNA	1		2	232-kb del 11-Mb del	2
18	NA08331	Coriell gDNA	1		2	2-Mb del 12-Mb del	2
19	NA09216	Coriell gDNA	1		2	110-kb del 17-Mb del	2
20	NA50180	Coriell gDNA	1		3	1.5-Mb del 28-Mb del 431-kb dup	3
21	NA04099	Coriell gDNA	3		1	131-kb del	3
22	NA05115	Coriell gDNA	3		1	164-kb del	3
23	NA09981	Coriell gDNA	3		1	295-kb dup	3
24	NA10636	Coriell gDNA	3		1	19-Mb dup	3
25	NA12606	Coriell gDNA	3		1	40-Mb dup	3
26	Individual_1	Blood	3		1	60-kb del	3
27	Individual_2	Blood	3		1	437-kb dup	3
28	Individual_3	Blood	3		1	378-kb dup	3
29	Individual_4	Blood	3		1	114-kb del	3
30	Individual_5	Blood	3		1	124-kb dup	3
		TOTAL	50	1	35		55

### DNA preparation

DNA was extracted from whole blood using either (i) the Circulomics Nanobind® HMW DNA Extraction Kit (PacBio, Menlo Park, California, United States) or (ii) the NEB Monarch® Genomic DNA Purification Kit (New England Biolabs, Ipswich, Massachusetts, United States). Modifications to the manufacturer’s instructions are described in Additional file 3. For all DNA samples, including DNA obtained directly from Coriell, the DNA concentration was checked with an Invitrogen™ Qubit™ 4 fluorometer. DNA was sheared using a Covaris g-TUBE™, with 50 µl of DNA transferred to a g-TUBE, and then centrifuged twice for two minutes at 9000 rpm, flipping between the two spins to yield target fragment sizes of 10–15 kb. The sizes of the resulting fragments were verified using a TapeStation 4150 (Agilent, Santa Clara, California, United States).

### Library preparation and sequencing

We used approximately 1.5-2 µg of DNA as starting material for library preparation using the Ligation Sequencing Kit (SQK-LSK110; Oxford Nanopore Technologies, Oxford, United Kingdom). Modifications to the manufacturer’s instructions are described in Additional file 3. Each prepared library was loaded onto a MinION flow cell (R9.4.1; FLOMIN-106D; Oxford Nanopore Technologies) and sequenced on a GridION Mk1 device (Oxford Nanopore Technologies) for 48 h, running MinKNOW v22.03.4. Adaptive sampling was configured using a custom BED file for each sample (see "BED file design" section below) using the Genome Reference Consortium Human Build 37 (GRCh37) as the reference genome [14]. We sequenced 30 unique samples (a total of 50 with replicates) on individual flow cells. Inter-run and intra-run replicates were included to test the robustness of our assay (Table 1 and Additional file 1: Tables S1 and S2).

### BED file design

For all samples, a custom BED file was created that targeted nine 500-kb regions as controls, for a total of 4.5 Mb of control target sequence (Additional file 1: Table S4). Because the CNV of interest differed in every sample, we created an additional target region for each sample that included the entirety of the expected CNV along with 500 kb upstream and downstream (pad regions), where possible. Pad regions were truncated if the start or end of the chromosome was less than 500 kb from the start or end of the CNV.

### Bioinformatics pipeline for CNV confirmation

The bioinformatics pipeline was implemented using Nextflow (Additional file 2: Fig. S1) and executed on an AWS EC2 instance (p3.2xlarge). Raw FAST5 files from the sequencer were converted to FASTQ files using Guppy v5.0.11 with the dna_r9.4.1_450bps_sup model (Guppy is available to ONT customers via their community site: https://community.nanoporetech.com). Minimap2 (v2.22) was used to align reads to the GRCh37 reference sequence [15]. Then, the samtools (v1.13) depth tool was used to calculate depth at all genomic positions, including off-target positions (using the -a flag) and excluding reads with a mapping quality of zero (using the -Q 0 flag) [16].

Using a custom R script, the read depth in the suspected CNV region(s) was compared with the read depth across five unaffected autosomal control regions; four of the original nine control regions were excluded because they were located on the sex chromosomes or displayed consistently low coverage (Additional file 1: Table S4 and Additional file 2: Figs. S2 and S3). A duplication or deletion was confirmed if the read depth across the suspected variant region was at least three standard deviations above or below, respectively, the mean depth of either the five genomic control regions or the five genomic control regions and the CNV-specific pad regions. The cut-off of three standard deviations was selected based on an initial analysis of a set of five samples sequenced on five initial development runs. To illustrate CNV confirmation, we generated regional mean depth plots for all CNVs of interest, where each plotted point denotes the normalized mean depth across a given control, pad, or CNV region. We also generated read-depth plots to visualize the depth across each target region, where the read depth was normalized by the mean read depth across all control regions. A detailed description of the pipeline is provided in Additional file 3.

To benchmark our approach, we assessed the ability of four existing long-read SV callers to detect the known CNVs in each of the samples. cuteSV, NanoVar, SVIM, and Sniffles were run on all 56 samples in our validation study using the recommended parameters [17–20]. A known CNV was considered to be detected by a caller if an SV was reported that matched the expected type (i.e. deletion or duplication) with start and end breakpoints within 1000-bp of the expected start and end locations.

To determine the depth required to achieve reliable CNV confirmation, we used the ‘samtools view --subsample’ tool to downsample BAM files to various depths, ranging from approximately 0.1X to approximately 15X, then ran our CNV confirmation method on the downsampled BAM files. We randomly generated 10 downsampled bam files at each depth. For this analysis, we used a sample with a 60-kb deletion (SM7481/Individual_1) and a sample with a 124-kb duplication (SM7419/Individual_5).

### Copy number

We used the mean depth ratio in the CNV region to interpret the copy number. For autosomes, the X chromosome in females, and pseudoautosomal regions (PARs): a deletion of one copy should have a normalized read depth of approximately 0.5; a deletion of both copies should have minimal to zero sequence data in the CNV region, and thus, a normalized read depth of approximately zero; and a duplication of one copy should have a normalized read depth of approximately 1.5, and each additional copy should add an additional 0.5 (e.g. 4 copies is 2, 5 copies is 2.5, etc.). For male X and Y chromosomes (except PARs on the X chromosome): A deletion should have minimal to zero sequence data in the CNV region, and thus, a normalized read depth of approximately zero; and a duplication of one copy should have a normalized read depth of approximately 2, and each additional copy should add an additional 1 (e.g. 3 copies is 3, 4 copies is 4, etc.).

### Confirmation of sample identity

We developed a method to perform sample matching between short- and long-read data using SNVs. This step served to demonstrate the utility of using SNVs to identify sample mix-ups and thus to ensure we had experienced no sample mix-ups. We used the five control target regions (Additional file 1: Table S4). Two control regions on chromosome 17, located close to one another, were treated as one region to eliminate the potential impact of linkage disequilibrium. We selected 150 gnomAD (r2.1.1) SNVs from each control region, with gnomAD allele frequencies between 0.4 and 0.6, to maximize the probability that the loci would vary between samples [21]. We determined the total number of reads that covered the position, number of reads with the reference allele, and number of reads with the alternate allele. For short-read data, an SNV was only included in the analysis if it was covered by at least ten reads. For long-read data, an SNV was only included if it was covered by at least one read. To account for low depth, we only included SNVs that were homozygous in both the short- and long-read data. A locus was considered “homozygous reference” if the reference allele frequency was greater than 0.95 and “homozygous alternate” if the alternate allele frequency was greater than 0.95. For each of the four control regions, we compared the genotype content of each sample – a region was considered a match between samples if greater than 90% of the genotypes matched across the region.

### ONT methylation analysis

We assessed methylation status in a Coriell sample known to have a duplication of chromosome 15q (NA22397). To enable the detection of CpG-related 5mC base modifications, raw FAST5 files from the sequencer were converted to FASTQ files using Guppy v6.4.2 with the dna_r9.4.1_450bps_modbases_5mc_cg_sup model. Reads were aligned to the GRCh37 reference sequence using minimap2 (v2.24) [15] by specifying the ‘--bam_out’ parameter to Guppy. Individual bam files were subsequently merged with ‘samtools merge’. Using a custom Python script with the Pysam package, we assigned each sequence read that intersected the *SNRPN* promoter CpG island region (chr15:25200035–25,201,054) to a haplotype using a nearby SNV (chr15-25199992-C-G) [16, 22, 23]. Sequence reads in the *SNRPN* promoter CpG island region were visualized using Integrated Genomics Viewer (v2.15.2); alignment color was set to indicate base modification (5mC).

## Results

### Adaptive sampling performance across samples

Across 50 replicate samples (from 30 independent samples), including 49 with known CNVs and one with an emulated false-positive CNV (Table 1), run on 50 MinION flow cells, we achieved an average on-target mean depth of coverage of 9.5X (range: 2.7X − 22.7X). Our average whole genome mean depth coverage was 1.13X; thus, we achieved a mean fold enrichment of our target regions of 8.4X (Additional file 1: Table S1). The average on-target read length across flow cells was 4805 bp, compared with the average whole genome read length of 603 bp (Additional file 1: Table S1). Our total target size varied from 5.51 Mb to 158.27 Mb. However, the mean on-target depth was not significantly affected by the total target size (Additional file 2: Figs. S4 and S5). We also observed that the mean on-target depth was significantly correlated with pore count on the flowcell at quality control (QC) check (Additional file 2: Fig. S6), but was not significantly impacted by library concentration (Additional file 2: Fig. S7).

### CNV confirmation with long reads

**Fig. 2:**
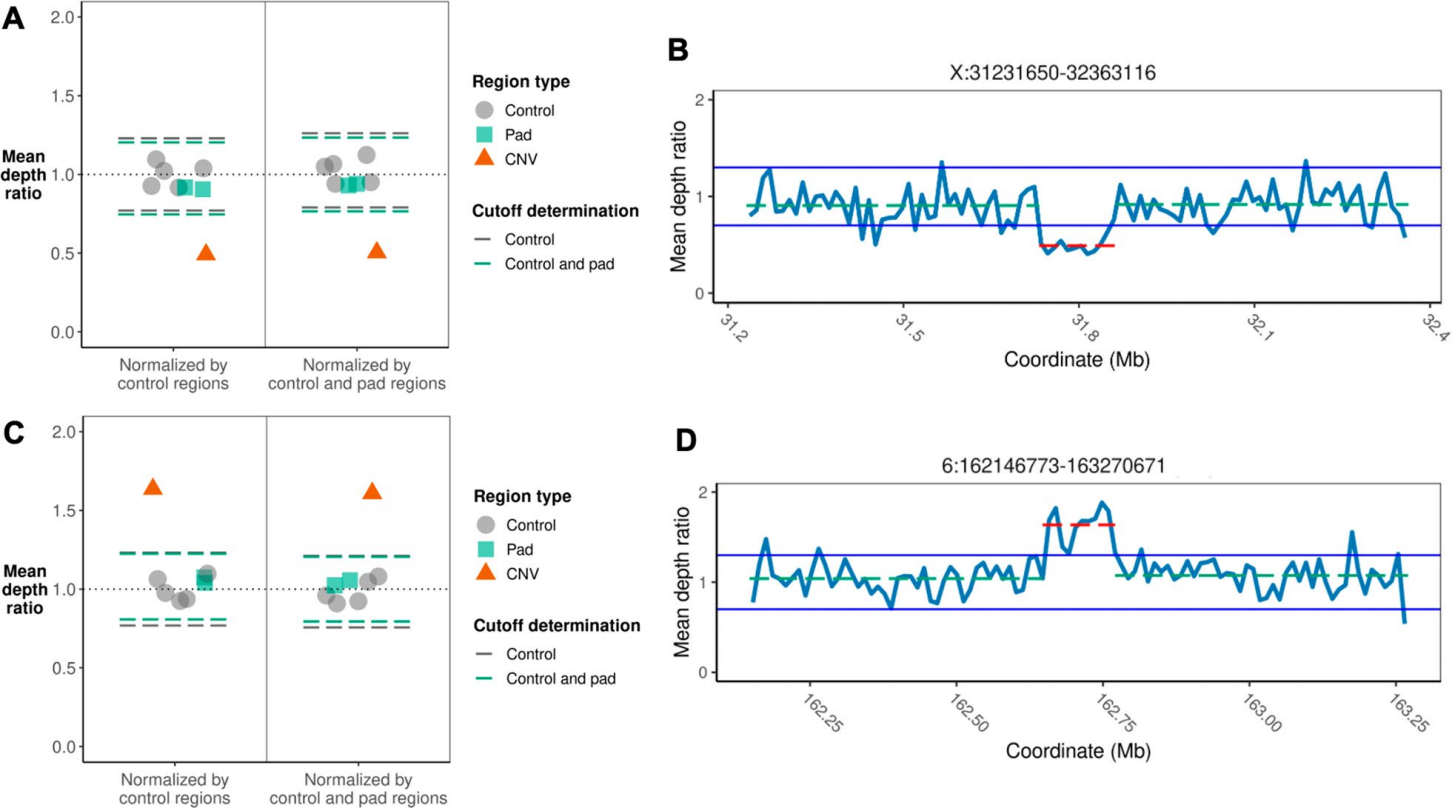
Examples of copy number variants (CNVs) identified by our pipeline. **A** Scatter plot of the regional mean depth for a Coriell sample (NA04099/SM4716) known to carry a deletion. A point was plotted for each of the five control regions, the two pad regions, and the deleted region. Each plotted point denotes the mean depth of the region normalized by either the mean depth across all control regions (left side of plot) or the mean depth across all control and pad regions (right side of plot). For each normalization approach, dashed lines are plotted to indicate three standard deviations from the mean of the normalized control regions (gray) and the normalized control and pad regions (green). **B** Read depth plot for a Coriell sample (NA04099/SM4716) known to carry a deletion. The mean depth in the deleted region was normalized by the mean depth across all control regions, calculated in non-overlapping windows that were 1% of the target size. The red dashed line indicates the mean depth ratio across the deleted region, and the green dashed lines indicate the mean depth ratios of the pad regions directly adjacent to the deleted region. **C** Scatter plot of the regional mean depth for a blood sample (Individual_5/SM7419) from a healthy individual known to carry a duplication. **D** Read depth plot for a blood sample (Individual_5/SM7419) from a healthy individual known to carry a duplication

**Fig. 3:**
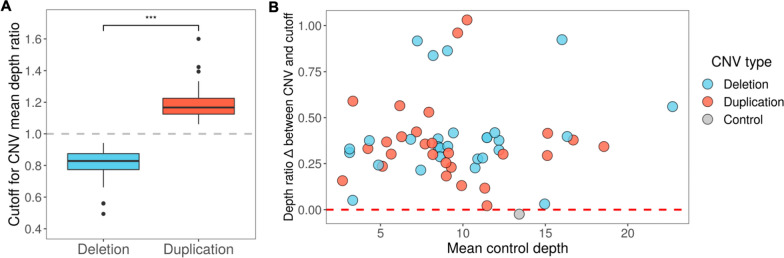
Cutoff thresholds for copy number variant (CNV) confirmation. **A** Boxplot displays of the cutoffs used to confirm deletions and duplications across 50 samples and 56 CNVs (including the false-positive CNV). The cutoff thresholds for CNVs were set at three standard deviations from the mean of either the mean depth ratios of the five control regions or the mean depth ratios of the five control regions and two pad regions, whichever was larger or smaller for deletions and duplications, respectively. ^***^, *p* < 0.001. The dashed gray line denotes a mean depth ratio of 1, which would be the expected mean depth ratio for a region not affected by a CNV. **B** Scatter plot of the difference between the CNV mean depth ratio and its dynamically determined cutoff for each of the 56 deletion, duplication, or control variants. The difference for deletions was calculated as the CNV depth ratio subtracted from the deletion depth ratio cutoff, and the difference for duplications was calculated as the duplication depth ratio cutoff subtracted from the CNV depth ratio, such that confirmed CNVs appear above zero, denoted by the red dashed line

The cutoff threshold for confirming CNVs was determined dynamically for each sample as three standard deviations above (for duplications) or below (for deletions) the mean depth of either the control regions or the control and pad regions (Fig. 2A and C). The median CNV depth ratio cutoff was 0.83 for deletions and 1.17 for duplications (Fig. 3A). Regardless of the mean on-target depth in the control regions, all deletions and duplications exceeded the cutoff threshold; thus, the presence of all CNVs was confirmed (Fig. 3B and Additional file 2: Fig. S8). Additionally, we examined read depth and, in most cases, observed an increase (for duplications) or decrease (for deletions) in depth across the CNV region of interest (Fig. 2B and D). In the false-positive sample, the mean depth in the region of interest was not three standard deviations from the mean depth of the control regions (Additional file 2: Fig. S9), and no change in depth was observed (Additional file 2: Fig. S10).

To assess the merits of our self-developed CNV confirmation method, we compared our results to four existing long-read SV detection tools: cuteSV, NanoVar, SVIM, and Sniffles. Correctly, all four of the SV callers did not detect a copy number change in the sample with the false positive CNV. However, all four SV callers achieved low sensitivity for known CNVs. Across the 55 known CNVs, cuteSV detected 2, NanoVar detected 5, SVIM detected 6, and Sniffles detected 14; the union across all four SV callers was 18 CNVs (Additional file 1: Table S5).

To determine the mean control depth required to achieve reliable CNV confirmation, we downsampled BAM files to various mean control depths to determine the depth at which we could consistently confirm the CNV in two samples with known microdeletions/microduplications. For sample SM7481, with a 60-kb deletion, we were able to consistently confirm the deletion at a depth of approximately 4X or greater, and for sample SM7419, with a 124-kb duplication, we were able to consistently confirm the duplication at a depth greater than approximately 5X (Additional file 1: Table S6).

### Copy number assessment

**Fig. 4:**
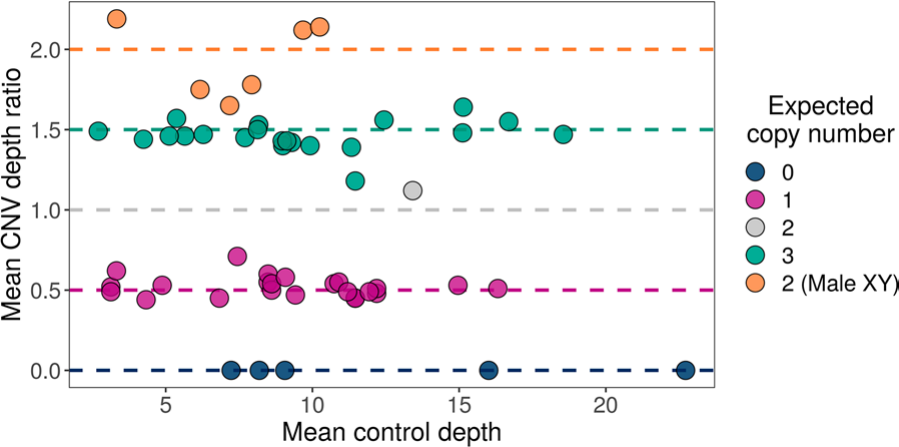
Scatter plot of the mean copy number variant (CNV) depth ratios for 56 CNVs (including the false-positive CNV), with differing expected copy numbers, across a range of mean control depths. The dashed horizontal lines indicate the expected mean depth ratio for each expected copy number

In addition to the presence of a deletion or duplication, we determined the number of copies gained or lost in each CNV region. Using the mean CNV depth ratio, we could determine whether there were 0, 1, 2, or 3 copies of a given genomic region (Fig. 4 and Additional file 2: Figs. S11 and S12). CNVs on the autosomes and female X chromosomes displayed a mean depth ratio of 0 and 0.5 when there were 0 copies or 1 copy of a region, respectively, and 1.5 when there were 3 copies of a region. The false-positive sample displayed a mean depth ratio close to 1, indicating the presence of 2 copies, as expected in a sample with no deletions or duplications on an autosomal chromosome. When a duplication was present on one of the sex chromosomes in a male sample, the mean depth ratio was approximately 2.

### Sample matching

**Fig. 5:**
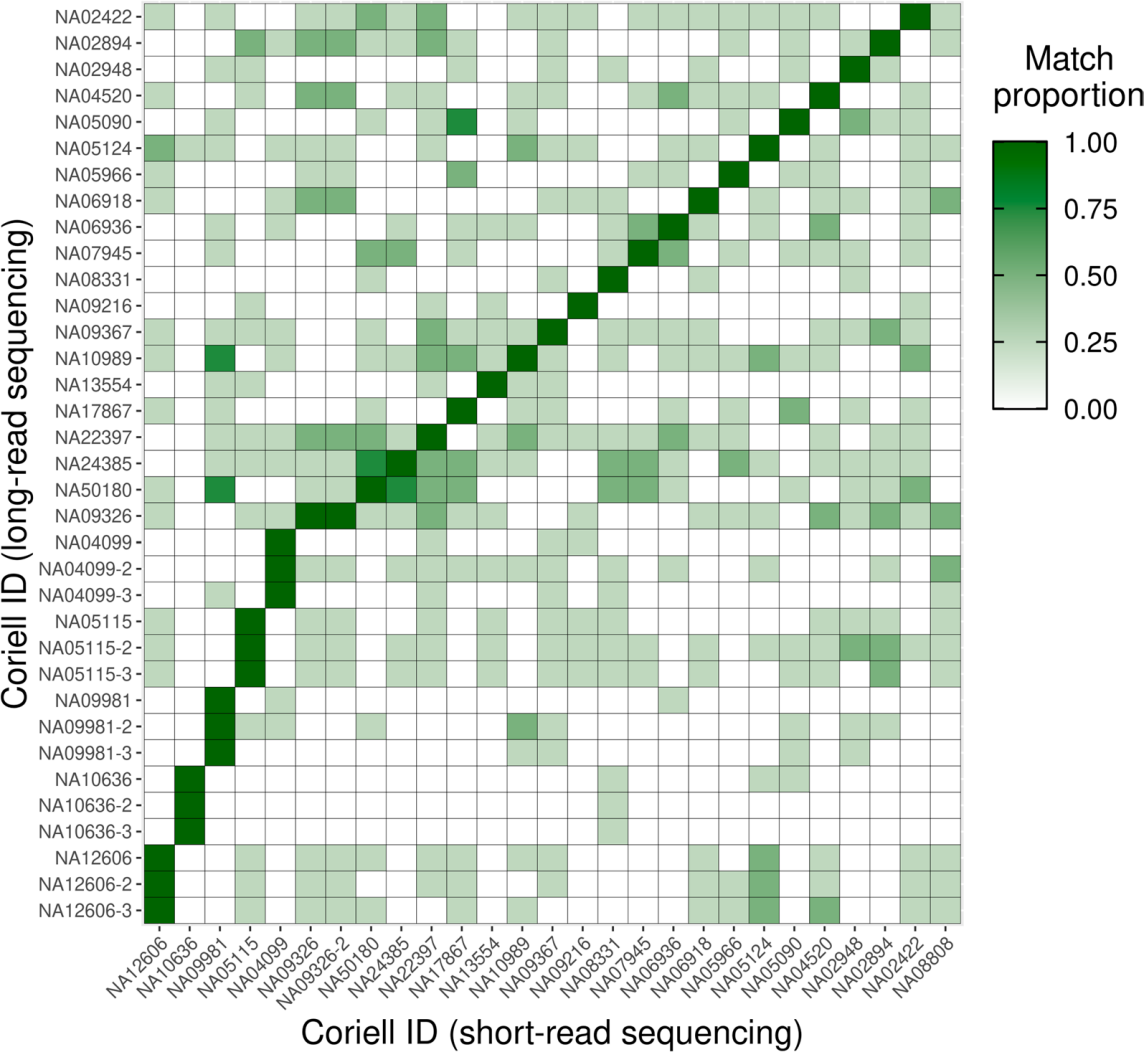
Heatmap of the proportion of four control regions that matched between Coriell samples sequenced previously with short-read sequencing and in the current study using long-read sequencing with adaptive sampling. The regions are matched based on single nucleotide variant content. The analysis also includes samples sequenced multiple times with long-read sequencing and short-read sequencing, as well as a sample sequenced using only short reads and thus with no long-read sequencing match

Because our workflow collects both short- and long-read data, we investigated an approach to ensure no sample mix-ups occurred. Building off of population allele frequencies in gnomAD and observed allele frequencies in our samples, we implemented a process to identify polymorphic sites and ensure no sample swaps occurred [21]. Using this method, we correctly assigned 26 unique Coriell samples (Fig. 5). We compared 27 short-read sequenced samples, including one replicate (NA09326), with 35 long-read sequenced samples, five of which were sequenced three times (NA12606, NA10636, NA09981, NA05155, and NA04099). In all cases, including replicates, the long-read data matched the expected sample in the short-read data. One Coriell sample (NA08808) was sequenced using only short-read sequencing and did not match any long-read samples, as expected.

### Methylation

**Fig. 6:**
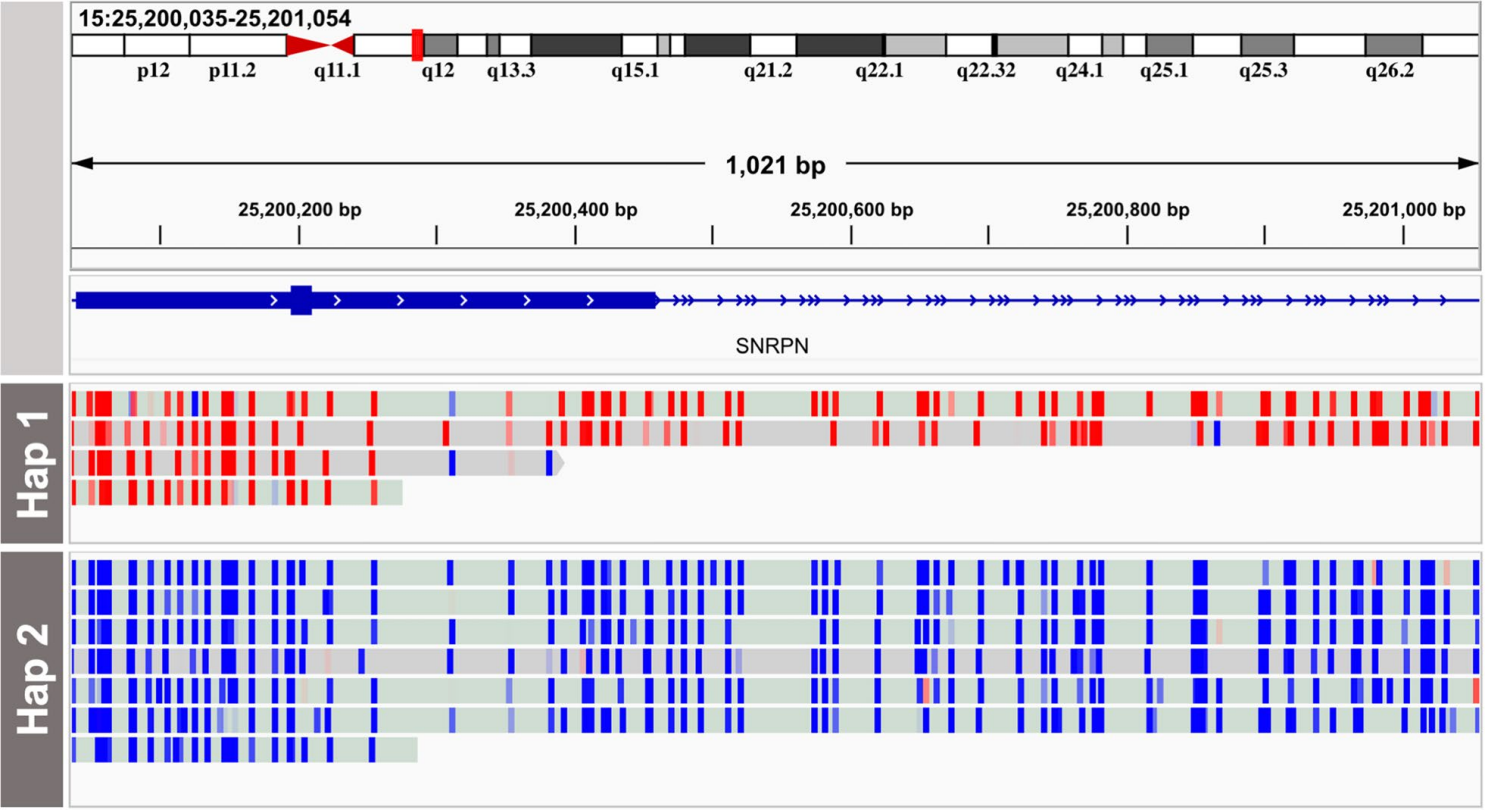
Integrative Genomics Viewer (IGV) snapshot of the *SNRPN* promoter CpG island region in Coriell sample NA22397, known to have a duplication of chromosome 15q. Reads assigned to haplotype 1 (Hap 1) are methylated (denoted by red) while reads assigned to haplotype 2 (Hap 2) are unmethylated (denoted by blue)

Using ONT read depth analysis, we confirmed a 5-Mb duplication (chr15:23565001–28602000) located on the q arm of chromosome 15 in Coriell sample NA22397. Chromosome 15q is known to harbor parent-of-origin methylation patterns, which have implications for patient prognosis when deletions or duplications occur in this genomic region. A specific CpG island located in the *SNRPN* promoter is methylated only on the maternally inherited allele [24]. We assigned long reads to a haplotype using a nearby SNV, located approximately 45 bp upstream of the CpG island (Additional file 2: Fig. S13). We observed 11 reads that intersected the *SNRPN* promoter CpG-island region; four of these reads were methylated, belonging to the presumed maternal haplotype (Fig. 6, Hap 1), and seven of the reads were unmethylated, belonging to the presumed paternal haplotype (Fig. 6, Hap 2).

## Discussion

We described an approach to confirm CNVs identified by traditional short-read sequencing using targeted Nanopore sequencing and assessed its performance across a panel of samples containing a variety of CNVs. We successfully confirmed all tested alterations in a blinded validation study, demonstrating its use as a confirmatory assay.

Nanopore sequencing offers several operational advantages over conventional methods for CNV confirmation following whole genome sequencing. The first is speed. The laboratory workflow can be immediately initiated following the identification of a variant of interest, without the need for the design and optimization of primers or additional reagents. This is possible because adaptive sampling computationally targets specific genomic regions during the sequencing run, with the sequencer dynamically accepting or rejecting fragments in real time based on sequence matching with a user-specified list of regions [10].

The second is flexibility. In this study, a unique genomic region in each of the samples was targeted by providing a computer file to the instrument, without the need for any additional assays or materials. In contrast, conventional methods for target enrichment, including hybrid capture, molecular inversion probes, and PCR, would have required designing assays and ordering reagents prior to sequencing the region(s) of interest. In five cases, we targeted more than one CNV and correctly detected each instance.

The third is the ability to detect and avoid sample swaps by comparing genotypes from an initial assay and a confirmatory assay. Ensuring sample identity is critical to clinical test integrity, and the method we developed conveniently uses data already generated by the CNV assays to informatically verify sample identity.

Long-read sequencing has advantages over short-read sequencing because it natively provides long-range haplotypes and CpG methylation status. Although additional read depth would be required to confidently assess the parent of origin of the duplicated allele, we have demonstrated that methylated reads can be phased such that parent-of-origin could be determined in samples with sufficient depth. Parent-of-origin for this variant has potential prognostic implications because individuals with 15q11.2-q13 duplications of the paternal allele typically develop less severe autism spectrum disorder than individuals with duplications of the maternal allele [25]. In the case we presented, we relied on a single nearby SNV to phase reads. To improve accuracy in future studies, we will increase the number of variants for phasing by expanding our analysis to include indels and by applying our method to sample data with longer read lengths which will span more heterozygous loci. The ability to informatically “reflex” from the initial assay streamlines this process. Other recent studies have demonstrated the advantage of long-read sequencing as a single test by showing that STRs can be characterized and phased in individuals with various neurologic diseases [11, 25].

The flexibility of this platform suggests that, alongside confirmation of a given variant of interest, additional regions of interest can be programmed to: (i) elucidate the “second hit” for individuals with one pathogenic variant in a gene with recessive inheritance [25]; (ii) screen for repeat expansions missed by conventional sequencing [26]; (iii) resolve complex SVs [27]; and (iv) identify epigenetic signatures associated with disease [28, 29].

Several areas for improvement remain. First, we observed substantial inter-run variability in the read depth between flow cells and samples. Although this variability did not affect our ability to confirm any CNVs, we plan to examine methods to predict variability and improve performance consistency. Second, the smallest CNV confirmed in our study was 40 kb, and we only attempted to validate deletions and duplications (i.e., no other SV classes: inversions, translocations, etc.). However, recent studies have demonstrated the ability of long-read sequencing to detect smaller SVs as well as those of other classes [9, 11]. Third, the cost of this long-read sequencing assay is higher than some alternative CNV confirmation methods including qPCR, although existing techniques typically require customization for each additional target region. We are currently exploring strategies to reduce the cost of our assay. As such, we have already observed increased read depth and quality with the latest R10.4 MinION flow cells, and are also investigating the ability of our assay to perform using multiplexed samples on higher throughput PromethION flow cells.

## Conclusions

First, across a cohort of 30 samples containing 35 unique CNVs ranging from 40 kb to 155 Mb, we achieved a diagnostic accuracy of 100%. Second, we used patient-specific single nucleotide variants from both short- and long-read data to determine sample identity as a safeguard against sample swaps. Finally, we extended our approach to determine methylation status and phase, thus enabling parent-of-origin testing in an individual with a chromosome 15q duplication syndrome, with prognostic implications. Importantly, these findings would typically require an additional assay, which would prolong time to diagnosis. Given our findings, we anticipate that targeted confirmation of short read-identified CNVs using ONT long-read sequencing with adaptive sampling represents an immediate and practical use of this powerful and flexible platform in clinical laboratories. Beyond its ability to inform disease management, this technology has the potential to broaden our understanding of the role of CNVs in disease.

## Supplementary Information

**Additional file 1. **Additional tables.

**Additional file 2. **Additional figures.

**Additional file 3. **Additional methods.

## Data Availability

Access to primary sequence data from participants of this study is controlled and cannot be made publicly available. All relevant results and summary data from this study are available in the supplementary files.

## Abbreviations

ACMG: American College of Medical Genetics and Genomics
bp: base pair
CMA: Chromosomal Microarray
CNV: Copy Number Variant
kb: kilobases
Mb: Megabases
NDD: Neurodevelopmental Disorder
NGS: next-generation sequencing
ONT: Oxford Nanopore Technologies
PAR: Pseudoautosomal Region
SNV: Single Nucleotide Variant
STR: Short Tandem Repeat
SV: Structural Variant
