# Supplementary material for: Implementation of Nanopore sequencing as a pragmatic workflow for copy number variant confirmation in the clinic
Source: J Transl Med. 2023 Jun 10;21:378. doi: 10.1186/s12967-023-04243-y (PMC10257846; doi:10.1186/s12967-023-04243-y)
Supplement: Supplementary file 2 — Additional file 2. Additional figures. [file 12967_2023_4243_MOESM2_ESM.pdf]

# Additional file 2

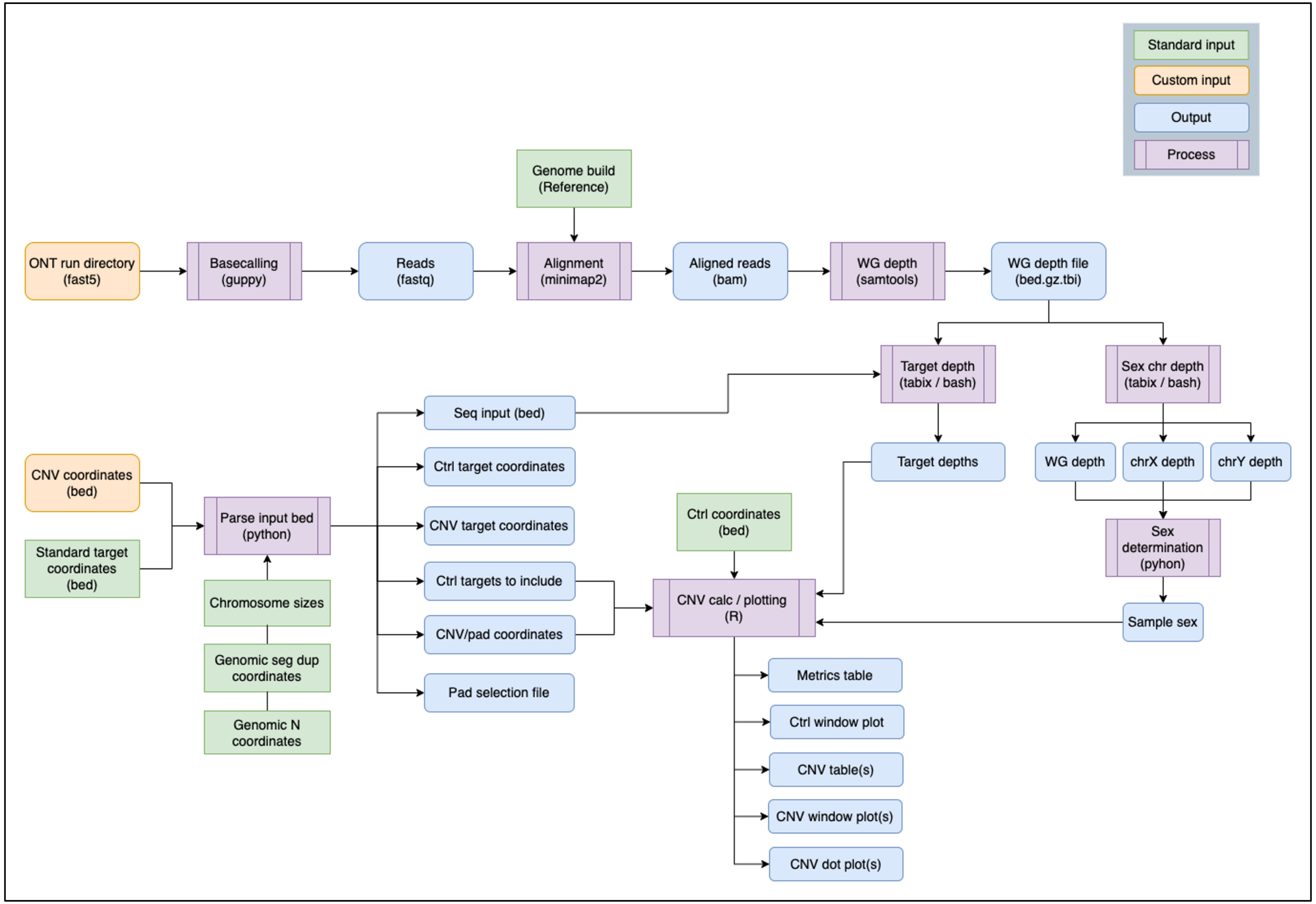


**Figure S1.** Overview of the bioinformatics pipeline, implemented in Nextflow.

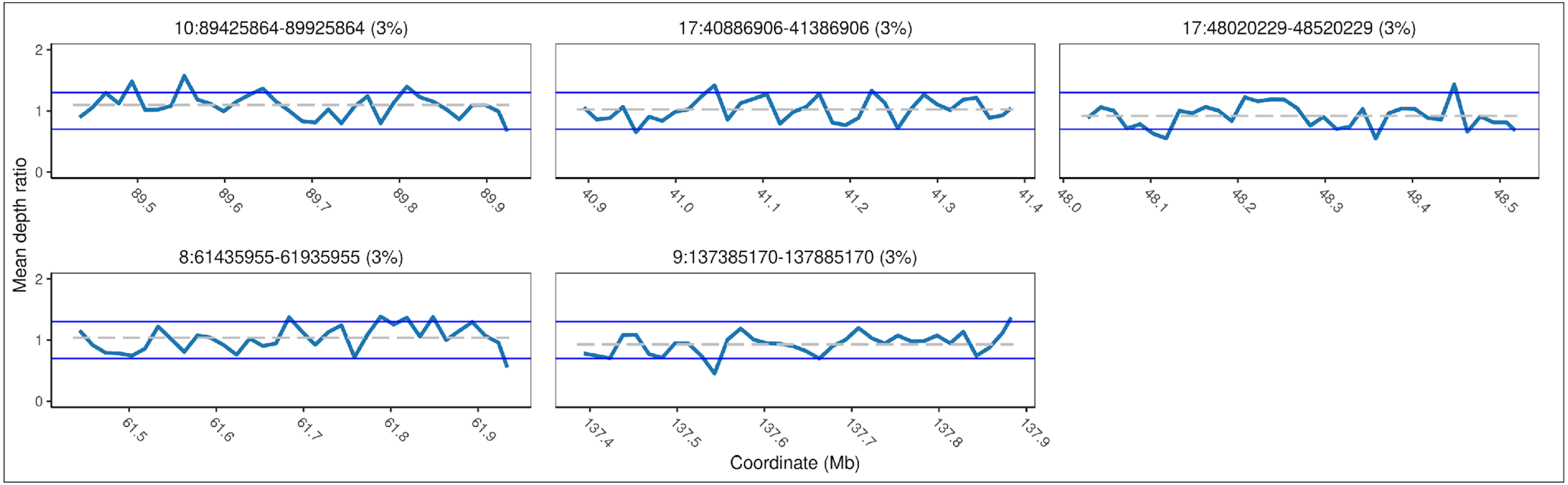


**Figure S2.** Read depth plots of the five control regions in Coriell sample NA04099/SM4716.

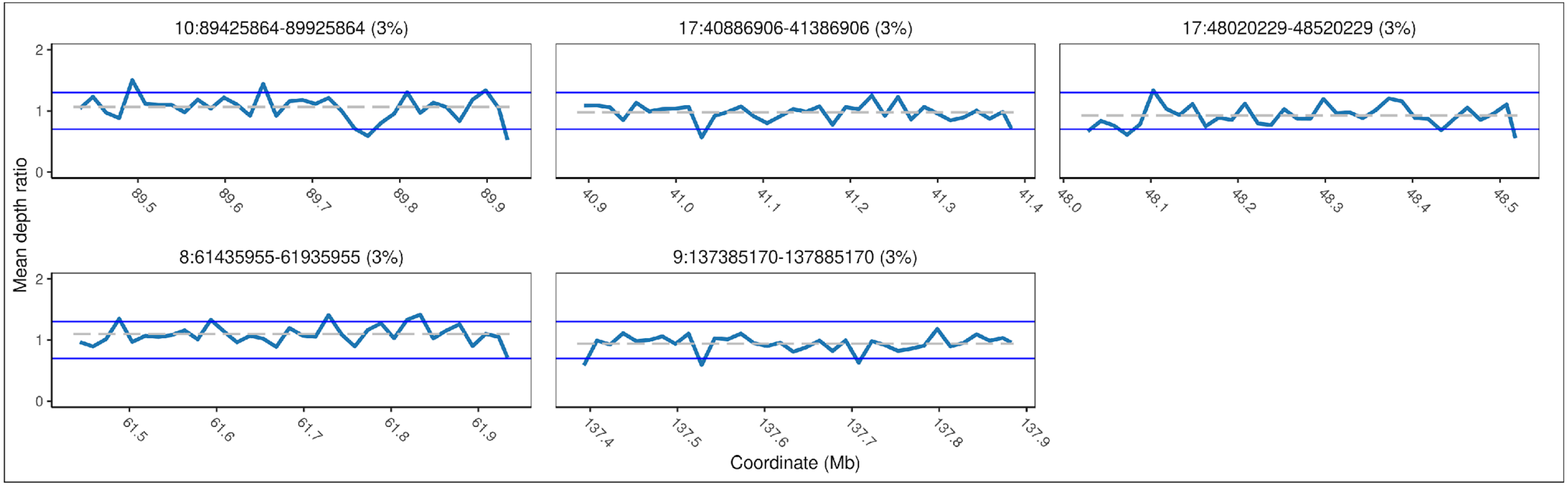


**Figure S3.** Read depth plots of the five control regions from blood sample SM7419.

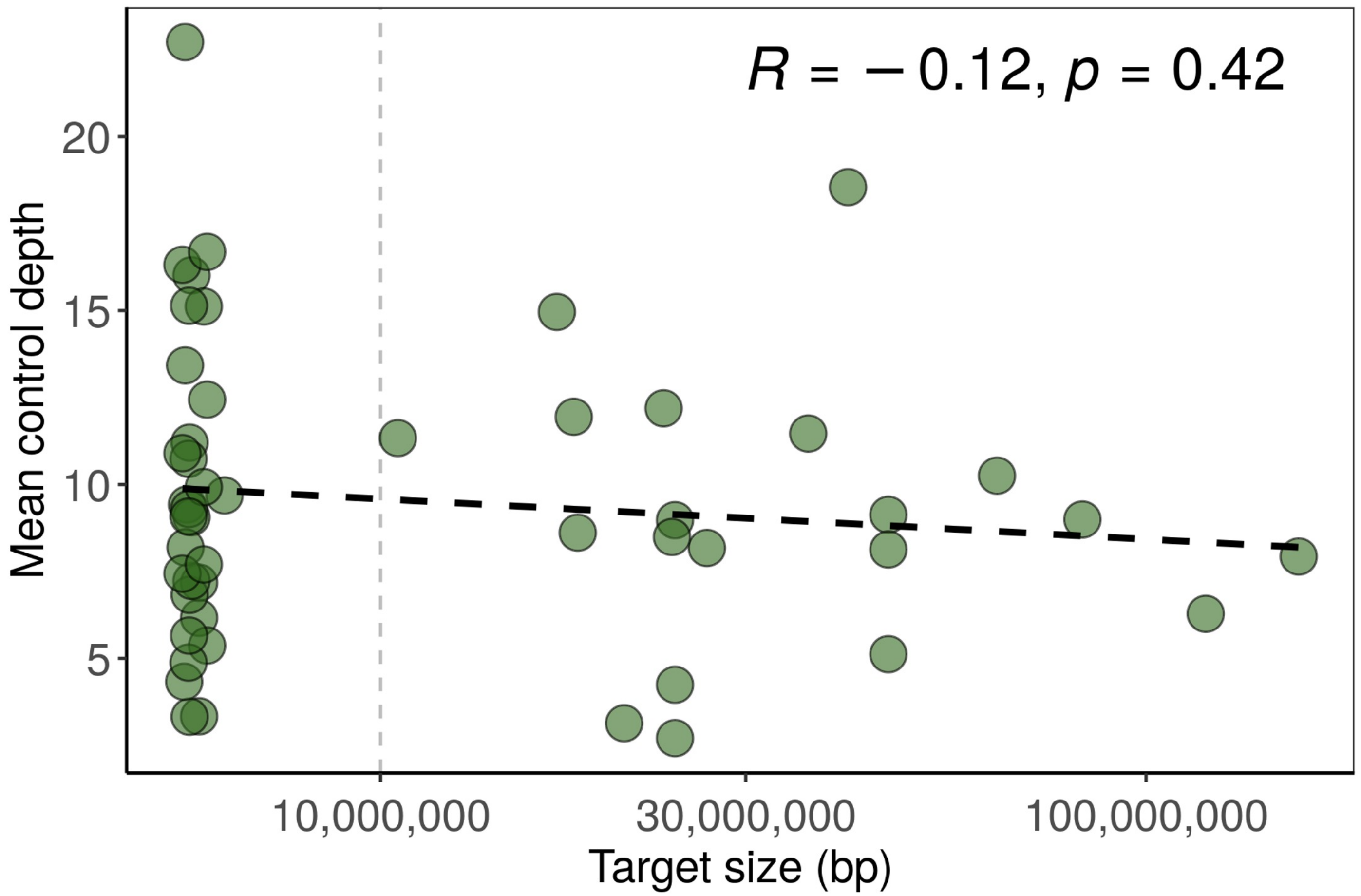


**Figure S4.** Scatter plot displays the relationship between the total size targeted with adaptive sampling and the mean depth of the targeted control regions across all 50 samples. The linear regression trendline is plotted, and the Pearson correlation coefficient and its associated *p*-value is indicated on the plot.

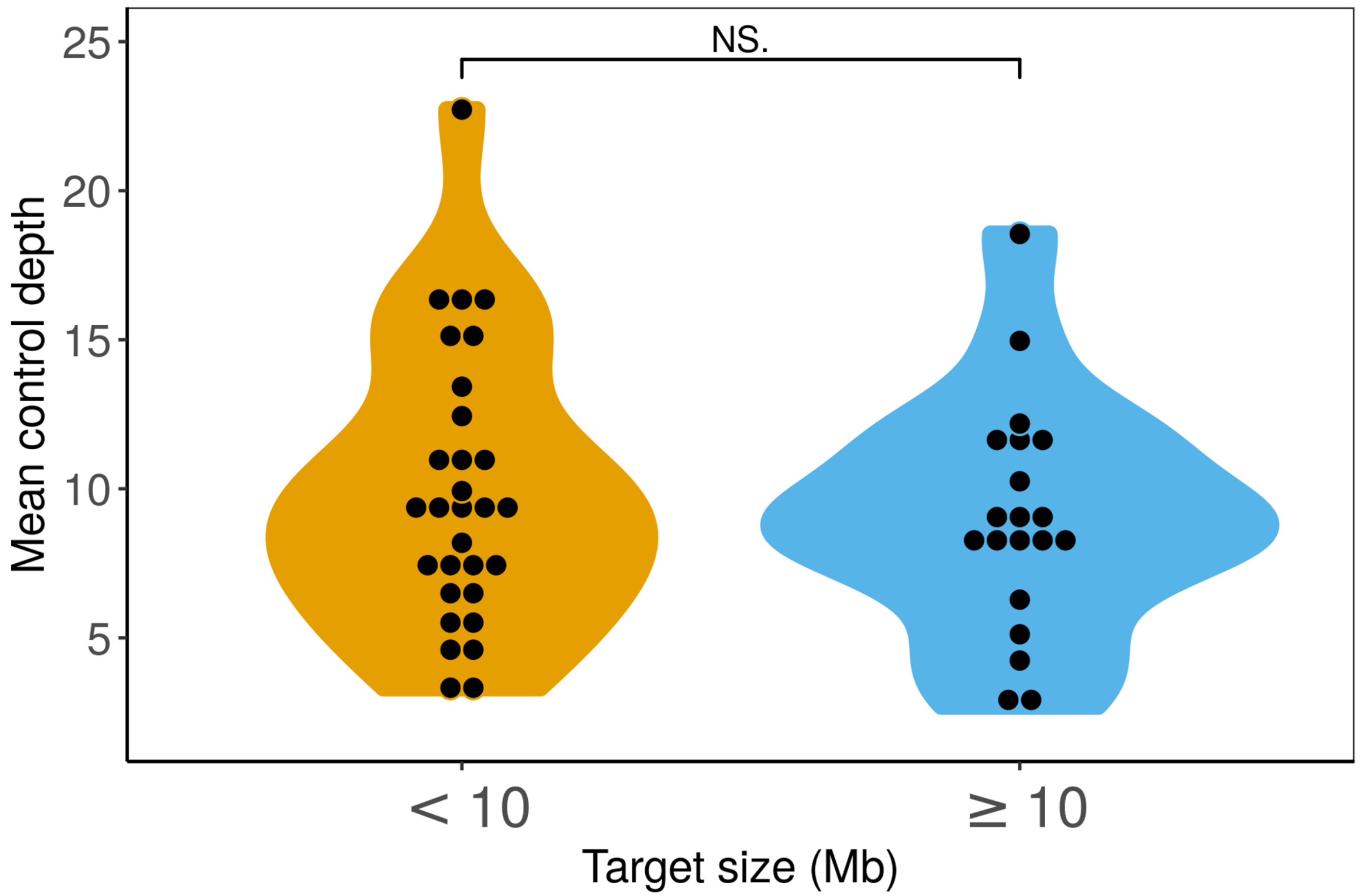


**Figure S5.** Violin plot displays the mean control depth when the total target size is less than 10 Mb ($N$=30) or at least 10 Mb ($N$=20). The significance level between the two groups is indicated.

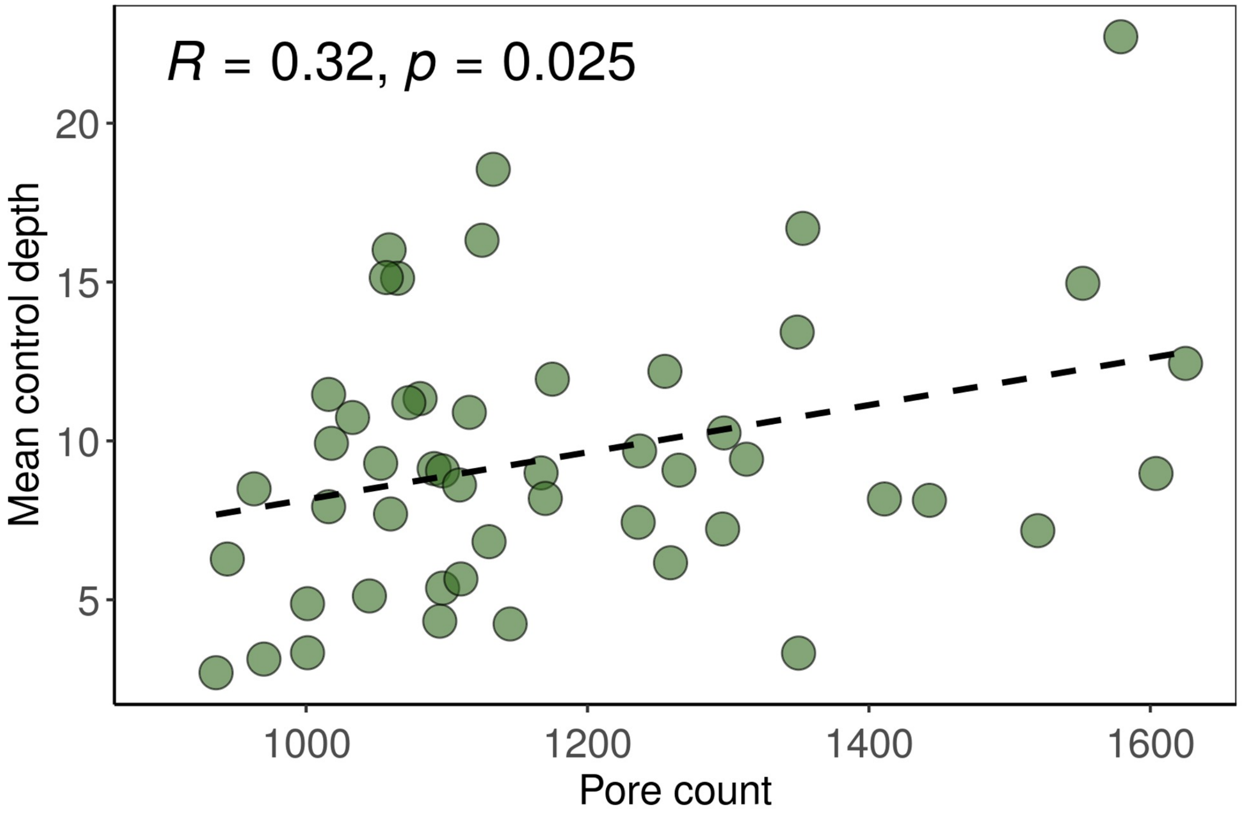


**Figure S6.** Scatter plot displays the relationship between the pore count on the ONT MinION flow cell at quality control (QC) check and the mean depth of the targeted control regions across all 50 samples. The linear regression trendline is plotted, and the Pearson correlation coefficient and its associated *p*-value is indicated on the plot.

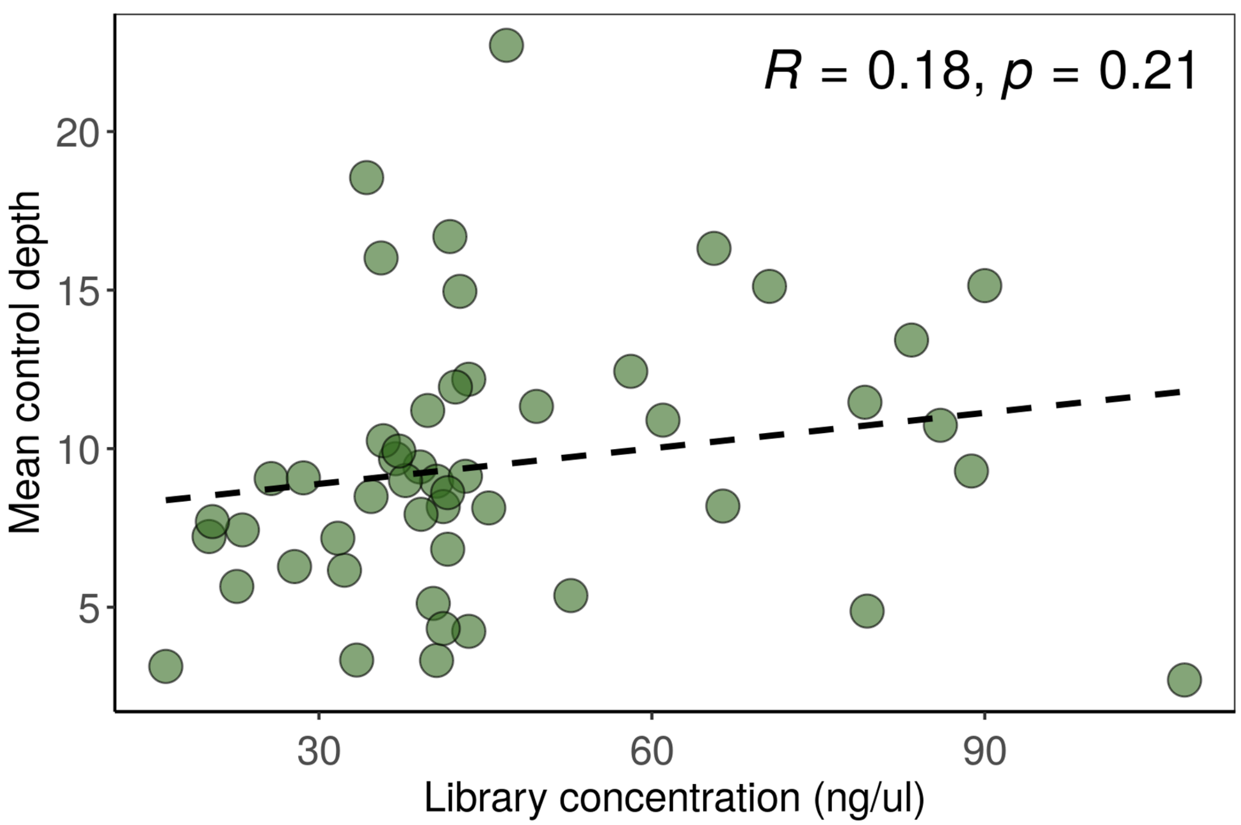


**Figure S7.** Scatter plot displays the relationship between the final library concentration and the mean depth of the targeted control regions across all 50 samples. The linear regression trendline is plotted, and the Pearson correlation coefficient and its associated *p*-value is indicated on the plot.

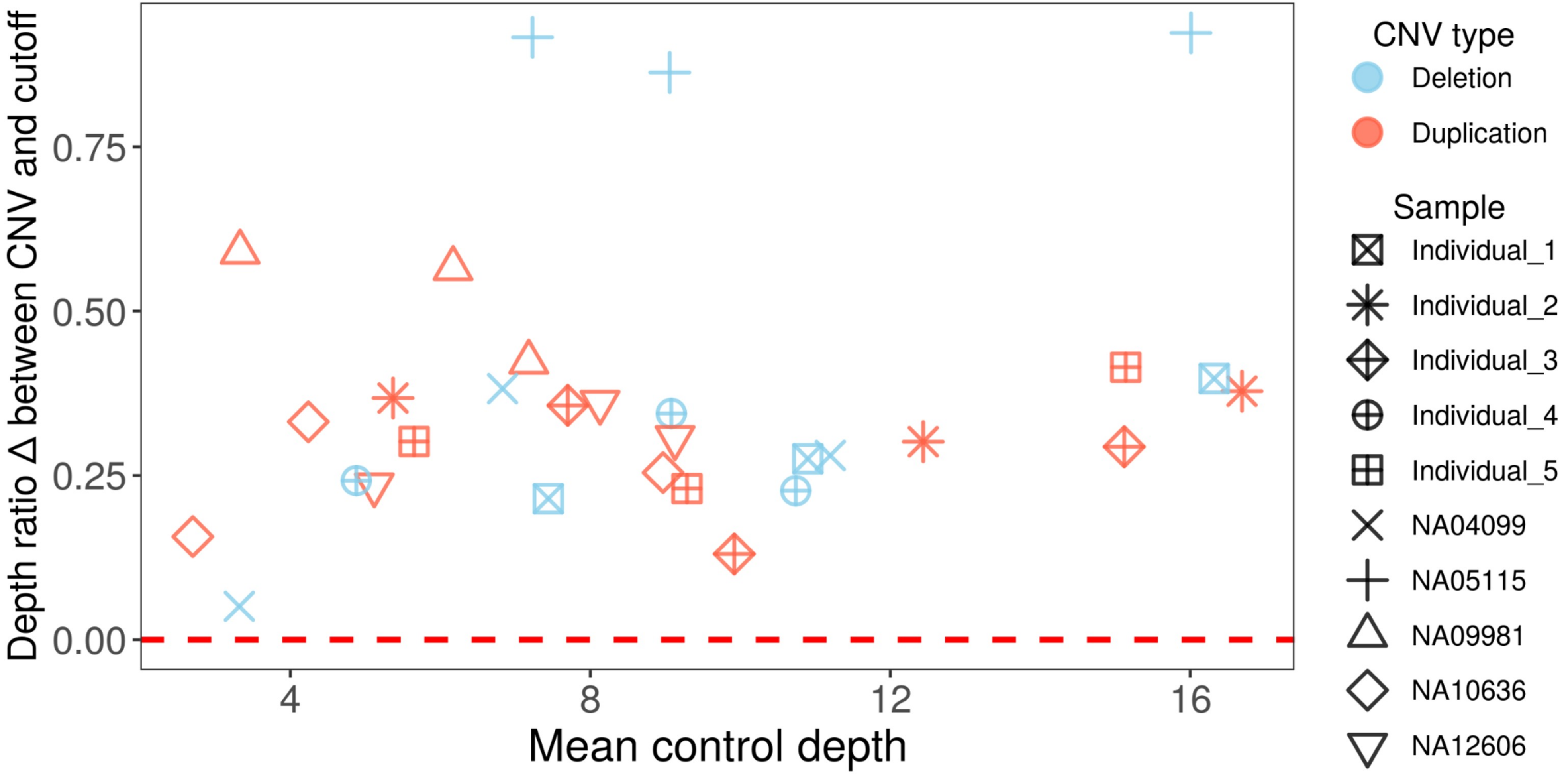


**Figure S8.** Scatter plot of the difference between the CNV mean depth ratio and its dynamically determined cutoff for each of the ten deletion or duplication variants assessed in triplicate. Replicates of the same sample/CNV are denoted with the same shape. The difference for deletions was calculated as the CNV depth ratio subtracted from the deletion depth ratio cutoff, and the difference for duplications was calculated as the duplication depth ratio cutoff subtracted from the CNV depth ratio, such that confirmed CNVs appear above zero, denoted by the red dashed line.

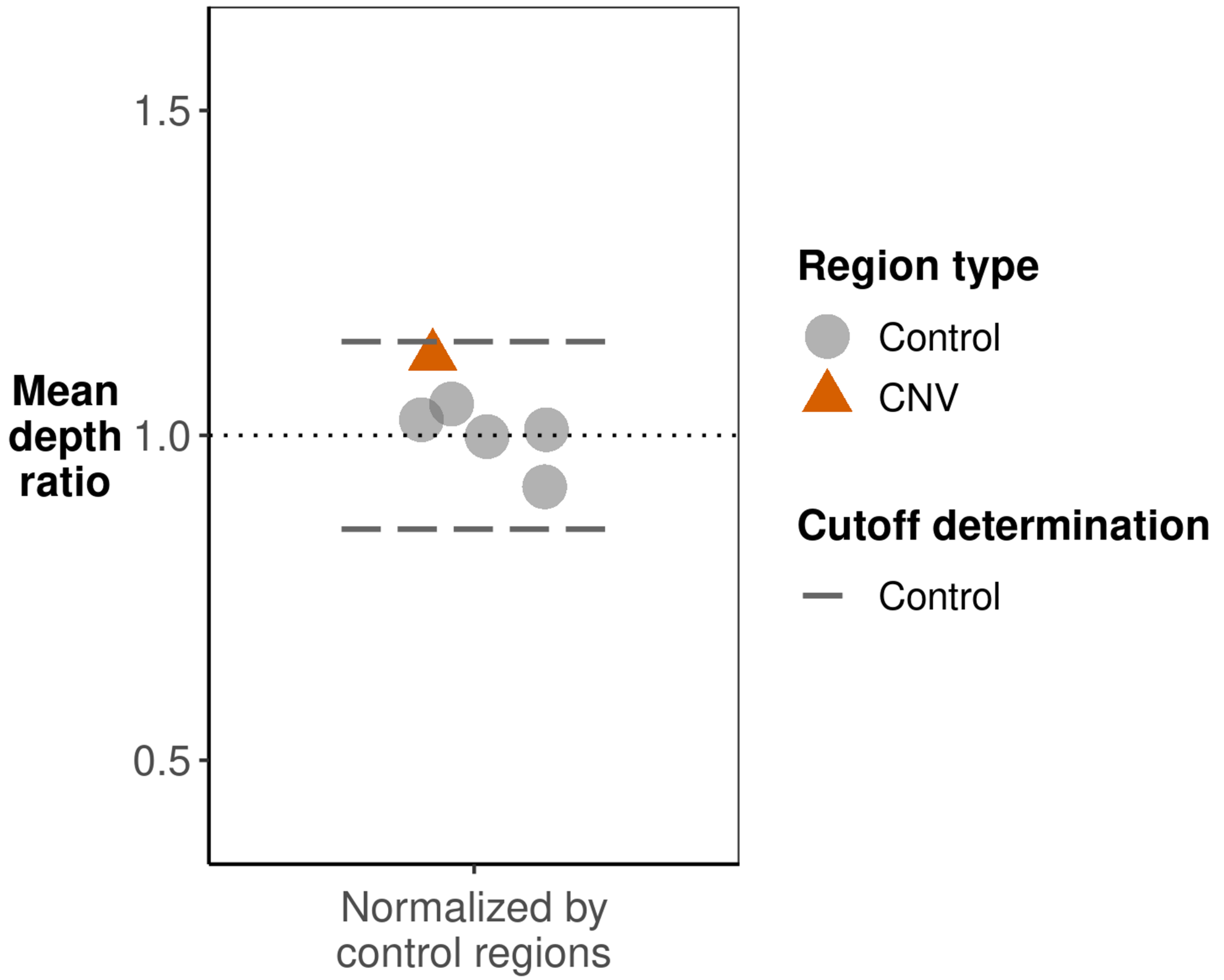


**Figure S9.** Scatter plot displaying the regional mean depth for Coriell sample NA24385/SM5004 with a known negative CNV call. A point was plotted for each of the five control regions and the “deleted” region. No pad regions were available for this CNV region.

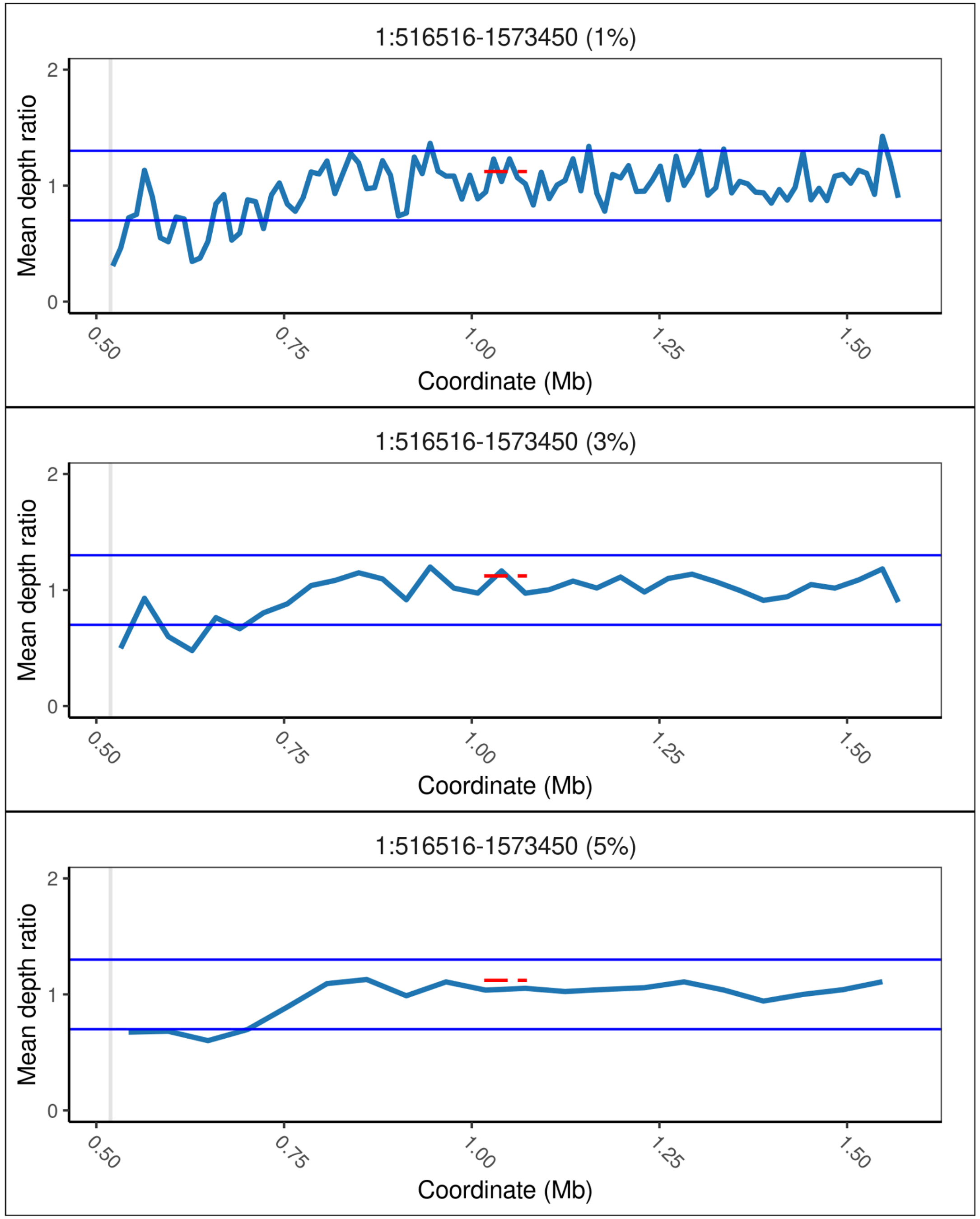


**Figure S10.** Read depth plot for Coriell sample NA24385/SM5004 with a known negative CNV displays the mean depth in the “deleted” region normalized by the mean depth across all control regions, calculated in windows that are 1% (top), 3% (middle) and 5% (bottom) of the target size. The red dashed line indicates the mean depth ratio across the “deleted” region.

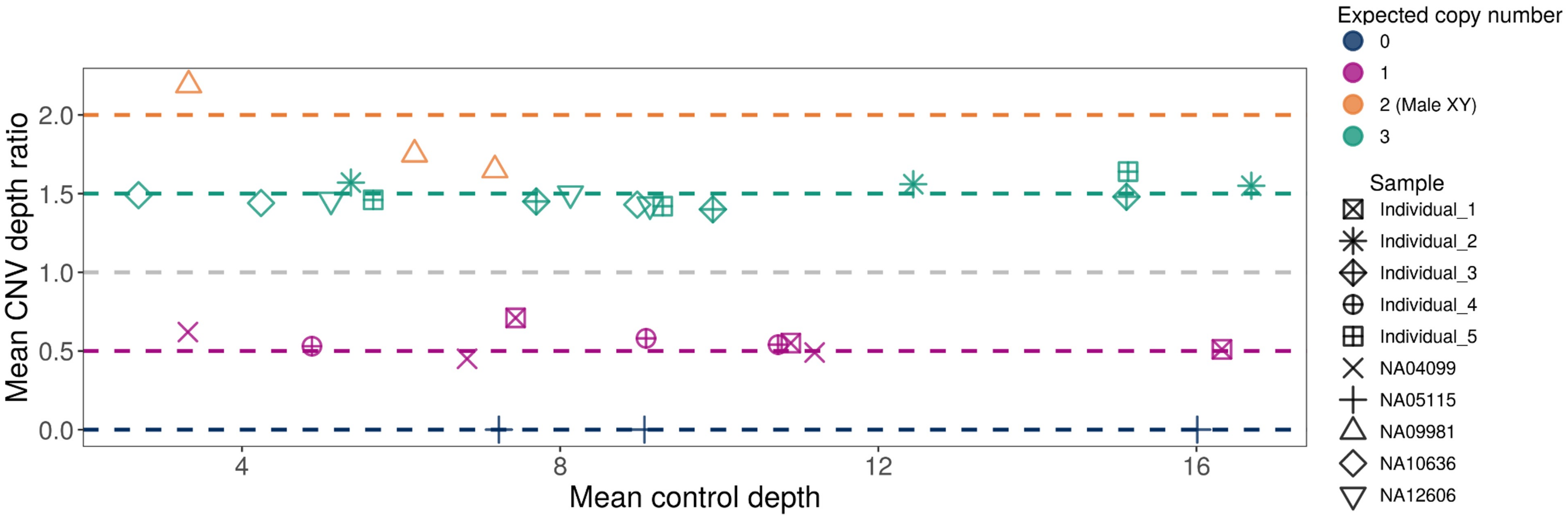


**Figure S11.** Scatter plot of the mean copy number variant (CNV) depth ratios for the ten CNVs assessed in triplicate. Replicates of the same sample/CNV are denoted with the same shape.The dashed horizontal lines indicate the expected mean depth ratio for each expected copy number.

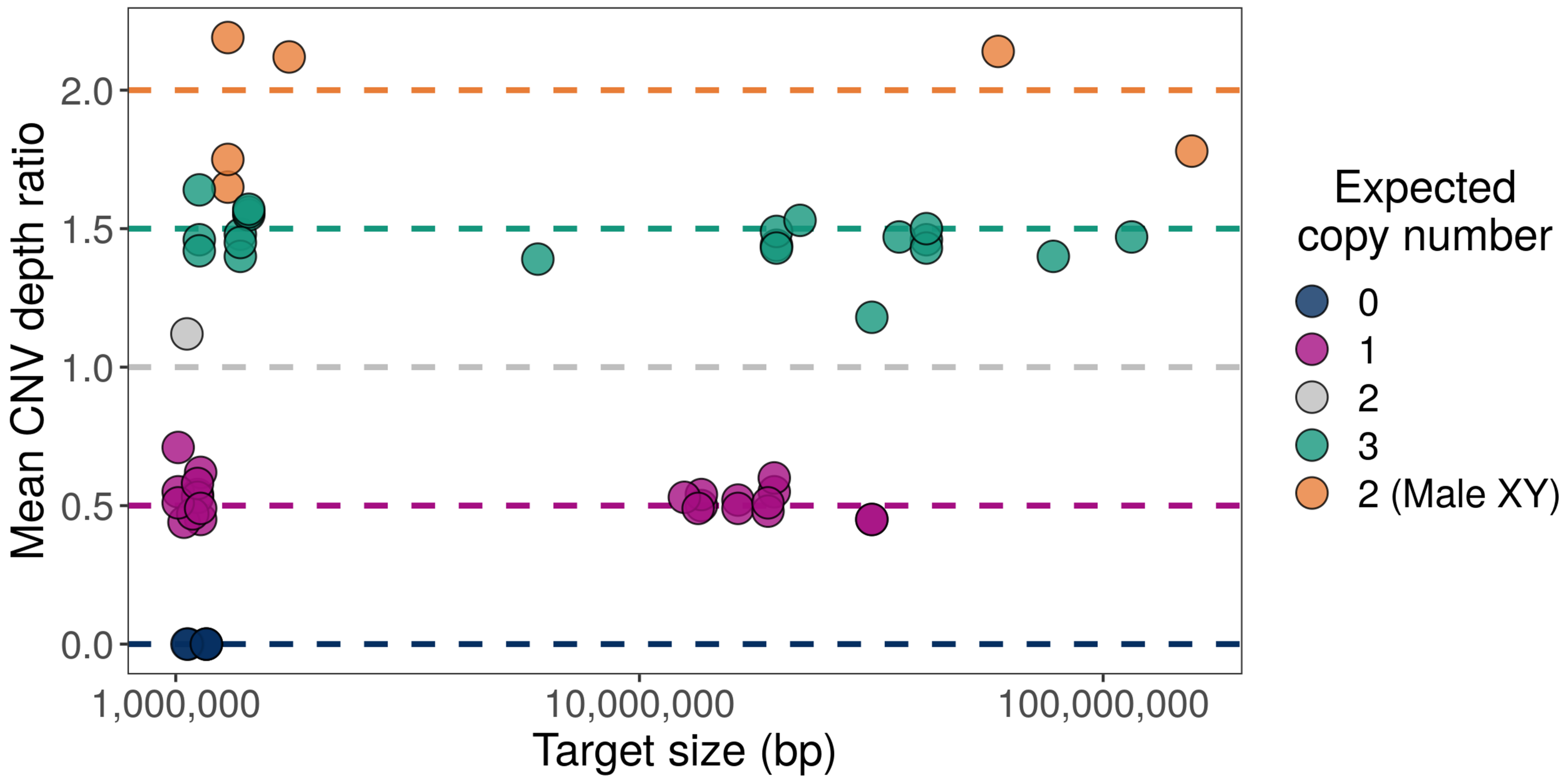


**Figure S12.** Scatter plot displays the mean CNV depth ratios across 56 CNVs, with differing expected copy numbers across a range of total target sizes.

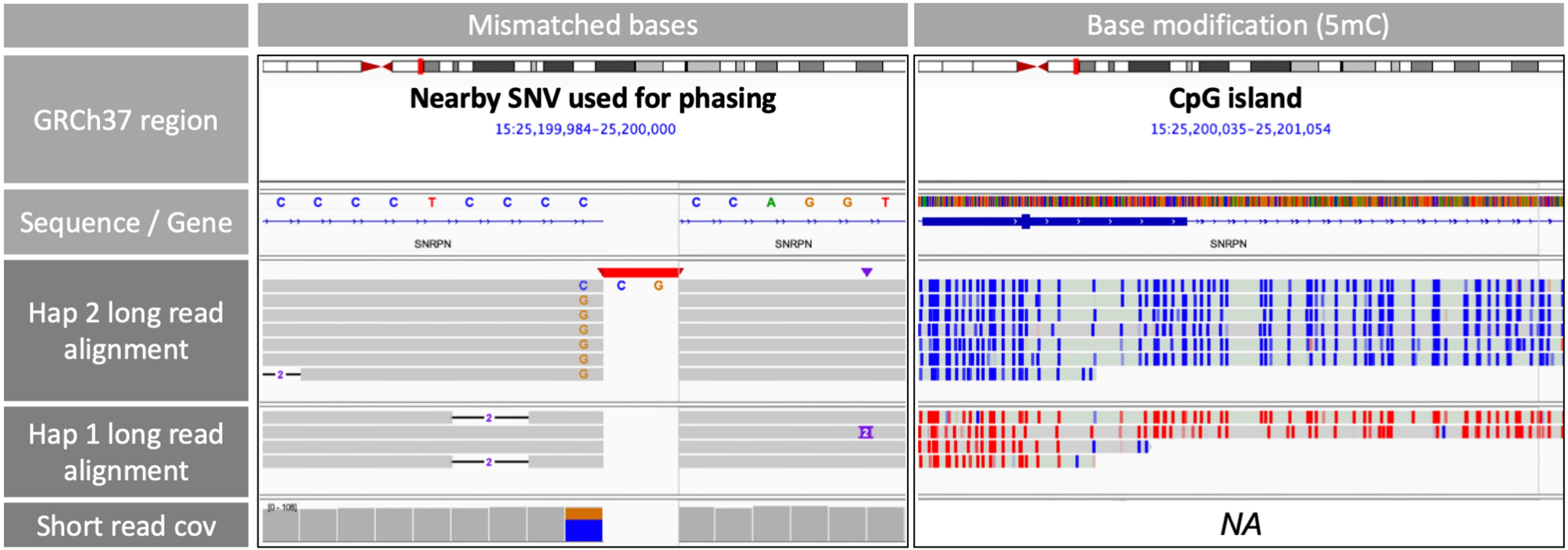


**Figure S13.** IGV snapshots display the phased methylation patterns in the *SNRPN* promoter region located in the 15q duplicated region of sample NA22397. Methylation patterns across reads are shown in the IGV snapshot on the right side of the figure. The SNV (chr15:25199992 C/G) used to phase the reads is displayed in the IGV snapshot on the left side of the figure. The SNV was also present in the short read data (bottom left panel). Reads with the reference allele (C) were assigned to haplotype 1 (middle left panel) and reads with the alternate allele (G) were assigned to haplotype 2 (top left panel). One read from haplotype 2 contained a 'CC' insertion error (denoted by the red insert).
