# Supplementary material for: Implementation of Nanopore sequencing as a pragmatic workflow for copy number variant confirmation in the clinic
Source: J Transl Med. 2023 Jun 10;21:378. doi: 10.1186/s12967-023-04243-y (PMC10257846; doi:10.1186/s12967-023-04243-y)
Supplement: Supplementary file 3 — Additional file 3. Additional methods. [file 12967_2023_4243_MOESM3_ESM.docx]

**Additional file 3: methods**

**DNA preparation**

We extracted DNA from the blood samples using either (i) the Circulomics Nanobind^®^ HMW DNA Extraction Kit (PacBio, Menlo Park, California, United States) according to the manufacturer’s instructions and eluting in 100 μl of Buffer EB or (ii) the NEB Monarch^®^ Genomic DNA Purification Kit (New England Biolabs, Ipswich, Massachusetts, United States) according to the manufacturer’s instructions and eluting in 100 μl of gDNA Elution Buffer, and with the following modifications: Frozen whole blood in EDTA was used. Frozen blood was thawed on the bench for 10 minutes. 400 μl of thawed blood was placed in a 1.5 mL microcentrifuge tube. Blood was microcentrifuged at maximum speed for 10 minutes. 300 μl of the blood supernatant was removed without disturbing the cell pellet at the bottom, leaving 100 μl in the tube as the starting material. After the initial elution of 100 μl per the manufacturer’s protocol, the elution step was repeated with 20 μl of warmed elution buffer to bring the final volume close to 120 μl.

**Library preparation and sequencing**

We used approximately 1.5-2 μg of DNA as starting material for library preparation with the Ligation Sequencing Kit (SQK-LSK110; Oxford Nanopore Technologies, Oxford, United Kingdom) according to the manufacturer’s instructions, with the following modifications: Adapter ligation reaction was incubated for 30 minutes instead of 10 minutes. If starting with less than 1.5 μg, the volume was doubled from 48 μl to 96 μl, and the reagents were doubled for the DNA repair and end preparation steps, but elution was performed using the volume indicated in the manufacturer’s instructions.

**Bioinformatic pipeline for CNV confirmation**

The bioinformatic pipeline was implemented in Nextflow (Additional file 2: Fig. S1) and executed on an AWS EC2 instance (p3.2xlarge). Raw FAST5 files from the sequencer were converted to FASTQ files using Guppy v5.0.11 with the dna_r9.4.1_450bps_sup model (Guppy is available to ONT customers via their community site: https://community.nanoporetech.com). Reads were aligned to the GRCh37 reference sequence using minimap2 (v2.22) [[20]](https://paperpile.com/c/NlzFVz/RhH7C). Then, the samtools (v1.13) depth tool using the -a flag was used to calculate depth at all genomic positions [[21]](https://paperpile.com/c/NlzFVz/n39JF).

The sex of the sample was determined from the read depth on the sex chromosomes. If the ratio of the Y chromosome mean depth to the whole genome mean depth was greater than 0.2, the sample was classified as male, otherwise the sample was classified as female (Additional file 1: Table S1).

Using a custom R script, read depth in suspected CNV region(s) was compared with the read depth across 5 unaffected autosomal control regions. The five regions used as control regions during post-sequencing depth analysis are indicated with a “Y” in the “control” column of Additional file 1: Table S4. We limited the control regions we used for analysis to those on autosomes without any known genome alignment issues.

A duplication or deletion was confirmed if the read depth across the suspected variant region was at least three standard deviations above or below, respectively, the mean depth of either the five genomic control regions or the five genomic control regions and the variant-specific pad regions.

To illustrate CNV confirmation, we generated regional mean depth plots for all CNVs of interest. A point was plotted for each control region, pad region (if available), and CNV region. Each plotted point denoted the mean depth of that region normalized by either (a) mean depth across all control regions or (b) mean depth across all control and pad regions. For each of conditions (a) and (b), dashed lines were plotted to indicate three standard deviations from the mean of (i) the normalized control regions and (ii) the normalized control and pad regions. The control and pad points were expected to appear between the dashed lines; however, the points for the pad regions could appear outside the control dashed lines due to imprecise CNV breakpoint coordinates detected from short reads. If a CNV was present, the CNV point was expected to appear above the dashed lines for a duplication or below the dashed lines for a deletion. If a CNV was not present, the CNV point was expected to appear between the dashed lines.

We also generated read depth plots to visualize depth across each target region, where read depth was normalized by the mean read depth across all control regions. Plots for all five control regions were generated to provide a reference point for the CNV(s) (Additional file 2: Figs. S2 and S3). Plots for all CNV regions were generated using multiple window sizes (i.e. 1%, 3%, and 5% of the CNV target region). If a CNV was present in the sample, we expected a change in depth at the location of the suspected CNV, and if a CNV was not present in the sample, we expected no change in depth.

If the sample was male and the CNV for confirmation was on one of the sex chromosomes (except for the pseudoautosomal regions (PARs) on the X chromosome), the mean depth for the CNV target region (including any pad regions) was multiplied by two, so that the mean depth ratio could be compared to the mean depth ratio in the control regions.

**Code availability**

For long-read sequence processing, the Guppy software was used to perform basecalling (Guppy is available to ONT customers via their community site: <https://community.nanoporetech.com>), followed by minimap2 (<https://github.com/lh3/minimap2>) to align sequence reads to the genome reference. Samtools depth was used to assess sequencing coverage across regions of interest (<http://www.htslib.org/>). Scripts for generating figures in this article to assess targeted sequencing and CNV confirmation performance, can be found at <https://github.com/myome/ont_cnv_confirmation_pub>.
